# Psychiatric hospitalisations for people who are incarcerated, 2009–2019: An 11-year retrospective longitudinal study in France

**DOI:** 10.1016/j.eclinm.2022.101374

**Published:** 2022-04-08

**Authors:** Thomas Fovet, Christine Chan-Chee, Maëlle Baillet, Mathilde Horn, Marielle Wathelet, Fabien D'Hondt, Pierre Thomas, Ali Amad, Antoine Lamer

**Affiliations:** a Univ. Lille, Inserm, CHU Lille, U1172 - Lille Neuroscience and Cognition, Lille F-59000, France; b Centre National de Ressources et de Résilience Lille-Paris (CN2R), Lille F-59000, France; c National Public Health Agency (Santé Publique France), Saint-Maurice F-94415, France; d Univ. Lille, Faculté Ingénierie et Management de la Santé, Lille F-59000, France; e Univ. Lille, CHU Lille, ULR 2694 - METRICS: Évaluation des Technologies de Santé et Des Pratiques Médicales, Lille F-59000, France; f Fédération Régionale de Recherche en Santé Mentale et Psychiatrie, Hauts-de-France, France

**Keywords:** Prison, Jail, Psychiatric hospitalisation, Forensic, Psychiatric hospital, Specially adapted hospital units, Data reuse

## Abstract

**Background:**

Despite the poor mental health status of people who are incarcerated, few studies have examined the number of psychiatric hospitalisations in this population. Since 2010, France has progressively opened nine full-time inpatient psychiatric wards exclusively for people who are incarcerated, called “specially adapted hospital units” (*unités hospitalières spécialement aménagées*, UHSAs, 440 beds). This study aimed to present the annual rates of psychiatric hospitalisations and primary psychiatric diagnoses among people who are incarcerated in France from 2009 to 2019.

**Methods:**

We used discharge reports from the French national hospital database to describe longitudinal retrospective administrative data of psychiatric hospitalisations for people in jail and prison between 2009 and 2019, the age, sex, and principal diagnoses of these patients, the proportion of voluntary versus involuntary care, and the interactions between UHSAs and other facilities.

**Findings:**

Between Jan 1, 2009, and Dec 31, 2019, 32,228 (92.2% men, *n* = 29,721; 7.8% women, *n* = 2 507) incarcerated people were hospitalised for psychiatric care (64,481 stays). The main diagnoses were psychotic disorders (27.4%), personality disorders (23.2%), and stress-related disorders (20.2%). The annual number of incarcerated people hospitalised in psychiatric care increased from 3263 in 2009 to 4914 in 2019. The gradual increase in the activity of UHSAs (300 hospitalisations in 2010 versus 3252 in 2019) was not associated with a reduction in the rate of hospitalisation of incarcerated people in local psychiatric hospitals.

**Interpretation:**

The creation of psychiatric hospitals specifically dedicated to the prison population has not stopped the hospitalisation of people who are incarcerated at psychiatric hospitals. These findings suggest that access to psychiatric hospitalisation remains problematic for people who are incarcerated in France.

**Funding:**

There was no funding source for this study.

**Research in context**

**Evidence before this study**

Despite the poor mental health status of people in prison, only a few cross-sectional studies have examined the number of psychiatric hospitalisations in this population. To appraise the current evidence, we searched PubMed to identify articles published from Jan 1, 1980 to Dec 31, 2021 using the key words “psychiatric hospitalisation”, “prison” and “incarceration”. In France, the government have built nine full-time inpatient psychiatric wards (440 beds) exclusively dedicated to people who are incarcerated, but no evaluation of the impact that the creation of these structures has had on the psychiatric hospitalisation of people who are incarcerated has been carried out.

**Added value of this study**

We examined the longitudinal retrospective administrative data of psychiatric hospitalisations for people in French jails and prisons between 2009 and 2019. Our results showed that the number of incarcerated people hospitalised in psychiatric care per year has substantially increased during this period. Importantly, the creation of these units was not associated with a reduction in the hospitalisations of incarcerated people in local psychiatric hospitals during the study period.

**Implications of all available evidence**

Planning psychiatric hospitalisations of people in prison is a challenging task, and our findings suggest that access to psychiatric hospitalisation remains problematic for people who are incarcerated in France. The creation of specialist units has improved the equivalence of psychiatric care inside and outside the walls of the prison by allowing voluntary hospitalisation for people who are incarcerated. Nevertheless, it has not led to a reduction in the rate of incarcerated individuals hospitalised in local psychiatric hospitals.

Alt-text: Unlabelled box

## Introduction

More than 11 million people are currently incarcerated around the world.^1^ The poor mental health status of this population has been extensively documented, and access to psychiatric care in correctional facilities is therefore a major public health issue.^2^ In France, the situation has been severely criticised in recent years.^3^, ^4^, ^5^ Indeed, in addition to harsh overcrowding conditions, a high prevalence of psychiatric disorders has been described in French prisons. A recent study found that 63.3% of 622 newly incarcerated people presented at least one psychiatric or substance use disorder identified with the Mini International Neuropsychiatric Interview (MINI).^6^ Compared with the general population, affective disorders, anxiety disorders, and psychotic disorders were approximately three times more frequent, and substance use disorders were eight times more prevalent.^6^ Overall, 41.6% of the sample had two or more psychiatric or substance use disorders.^6^ Before this study, Falissard et al. had already shown that 36% of people in prison have at least one psychiatric illness considered “marked” to “severe” (i.e., a rating of 5–7 on the *Global Clinical Impressions Scale*).^7^ Despite this alarming situation, very few studies have investigated the number of psychiatric hospitalisations in this population.^8^

The legal regulations for psychiatric treatment of people who are incarcerated diagnosed with a psychiatric disorder are quite different throughout the world.^9^ The French mental health care system for people in prison is based on three levels of care (see Table 1) that are the same regardless of the type of correctional facility. This care is fully affiliated with the public health system.^10^ Regarding full-time psychiatric hospitalisation, only involuntary hospitalisation was possible for people in prison until recent changes in the law.^11^^,^^12^ Almost all of these hospitalisations occurred in psychiatric hospitals without any additional security measures by the police. Occasionally, patients were referred to maximum-security wards (called *unités pour malades difficiles*, UMDs). This situation was associated with several issues in psychiatric hospitals, particularly the inappropriate use of isolation and mechanical restraint.^4^^,^^13^ In this context, the conditions for psychiatric full-time hospitalisation of people who are incarcerated have undergone significant changes, and new facilities called specially adapted hospital units (*unités hospitalières spécialement aménagées*, UHSAs) have been created in recent years.

**Table 1:** Description of the 3 levels of the French mental health care system for people who are incarcerated.

	Type of psychiatric care	Location	Voluntary* or involuntary⁎⁎ care	French designation
*Level 1*	Consultation and ambulatory care unit inside the prison	78 prisons (100% of French prisons)	Only voluntary	Health unit in prison *Unité sanitaire en milieu pénitentiaire* (USMP)
*Level 2*	Daytime hospital psychiatric beds inside the prison	26 prisons (14% of French prisons)	Only voluntary	Regional medical-psychological service *Service medico-psychologique regional* (SMPR)
*Level 3*	Psychiatric full-time hospitalisation	Local psychiatric hospitals	Only involuntary	No specific designation
		9 full-time inpatient psychiatric wards exclusively for people who are incarcerated (440 beds for incarcerated men, women, and juveniles)	Voluntary or involuntary	Specially adapted hospital unit *Unité hospitalière spécialement aménagée* (UHSA)
		10 maximum-security psychiatric units designed to accommodate patients (incarcerated or not) who “present such a danger to others that the necessary care, supervision and safety measures can only be carried out in a specific unit”. (620 beds for men/36 beds for women).	Only involuntary	Unit for difficult patients *Unité pour malades difficiles* (UMD)

⁎ care undertaken only with the consent of the person being treated.

⁎⁎ care undertaken without the consent of the person being treated, only in certain circumstances provided for by French law.

The nine UHSAs that have opened progressively in France since 2010 (with a current total capacity of 440 beds) are full-time inpatient psychiatric wards exclusively for people who are incarcerated.^14^^,^^15^ In these facilities, the prison administration ensures the security of the institution, manages entry/exit, and coordinates the transfer of patients. The UHSAs were designed both to reduce admissions of people who are incarcerated to psychiatric hospitals and to allow these people to be hospitalised with fewer restrictions of human rights. Voluntarily or involuntary hospitalisations are possible in UHSAs, unlike psychiatric hospitals, where people who are incarcerated can only be hospitalised involuntarily. Both types of hospitalisations are requested by the psychiatrist working in the prison. Involuntary hospitalisations are strictly regulated by law and are used when the individual who is incarcerated requires immediate inpatient care and constant supervision because of a mental disorder that makes it impossible for the individual to consent and that poses a danger to self or others.^16^

Since the first UHSA was built in 2010 in Lyon, only one cross-sectional study has been conducted to examine the activity of the UHSAs.^17^ This study showed that 4392 people who are incarcerated (7027 admissions) were hospitalised in psychiatric care in 2016 in France. Importantly, 1944 people were hospitalised in UHSAs, and 1787 people were still admitted to psychiatric hospitals despite the creation of these new facilities. However, in the absence of any longitudinal data, it is extremely difficult to determine the impact of the creation of UHSAs on the proportion of people who are incarcerated admitted in psychiatric hospitals and other facilities.

This 11-year retrospective longitudinal study (2009–2019) aimed to estimate the number of psychiatric hospitalisations for people who are incarcerated in France, stratified by calendar year and type of facility. We hypothesised that the creation of UHSAs decreased the admissions of people who are incarcerated to general psychiatric beds. The secondary objectives were to present patients’ characteristics, including age, sex, and clinical diagnoses, stratified by type of facility, across the study period of 2009–2019.

## Methods

### Study design

This retrospective study was performed in accordance with the *REporting of studies Conducted using Observational Routinely collected health Data* (RECORD) statement, which completes the *Strengthening the Reporting of Observational Studies in Epidemiology* (STROBE) statement when conducting retrospective observational studies.^18^^,^^19^

### Database

We used the *Programme de médicalisation des systèmes d'information* (PMSI) database, which includes individual-level data about the date of admission, length of stay, hospital code number, sector code, and outcome (i.e., discharge, hospital transfer, death) for all inpatient stays in psychiatric hospitals in France. The sociodemographic data from these records include sex, age, and place of residence. The principal diagnosis, defined as the main reason for admission, and the associated diagnoses related to comorbidities are documented in the PMSI database according to the French version of the International Statistical Classification of Diseases and Related Health Problems, 10th Revision (ICD-10). A unique national identification number for each patient allows the data from all hospital stays for the same patient to be linked.

Ethical approval was not needed for the present study because we had access to an anonymous administrative database. Moreover, the national French Public Health Agency (*Santé Publique France*) legally allows full access to national hospital discharge databases,^20^ including the PMSI, which is widely used for research purposes.^21^ The authors assert that all procedures contributing to this work comply with the ethical standards of the relevant national and institutional committees on human experimentation and with the Helsinki Declaration of 1975, as revised in 2008.

### Study population

We conducted a retrospective cohort study, and we included data related to all psychiatric hospitalisations for people who are incarcerated in France between 2009 and 2019. We did not distinguish between people incarcerated in *jails* (67%), which house people on remand or people with a residual sentence below two years, and people incarcerated in *prisons* (33%), which house people with long sentences. In this article, we used the term “prison” to refer to both prisons and jails.

Cases were identified through the following criteria:

- the hospital code number corresponding to UHSA, UMD, or daytime hospital psychiatric beds inside the prison (*Service medico-psychologique regional*, SMPR);
- the specific legal mode used for the involuntary hospitalization of people who are incarcerated (“D398”);
- the types of care (full-time or daytime hospitalisation);
- the specific sector code used for psychiatric facilities in prison (“P”).

As no data on level 1 (consultation and ambulatory care) are documented in the PMSI, only data on levels 2 (daytime hospitalisation in the *Service medico-psychologique regional*, SMPRs) and 3 (full-time hospitalisation in the psychiatric hospitals, the UHSAs, or the maximum-security units, UMDs) were included in this study (see Table 1). We used the term “psychiatric hospitals” to refer to both psychiatric hospitals and psychiatric wards of general hospitals.

Unlike SMPRs and UHSAs, which can only accommodate people who are incarcerated, maximum-security units (UMDs) and psychiatric hospitals can accommodate both incarcerated and nonincarcerated people. For this study, we only included data from people who are incarcerated. As they are not considered incarcerated in France, people who cannot stand trial due to mental illness and people found not criminally responsible on account of a mental disorder were not included in the present study.

Finally, additional indicators of psychiatric hospitalisations were collected for nonincarcerated people from the PMSI database for comparison purposes.

### Variables

We used several variables from the PMSI database: age and sex, date of admission, outcome (i.e., discharge, hospital transfer, death), principal diagnosis, type of psychiatric facility, and legal mode (voluntary versus involuntary care).

### Statistical analysis

Qualitative variables are presented with absolute numbers and percentages. Quantitative variables are presented as the median and interquartile range (IQR). The results are illustrated using line charts. The regression lines result from the affine function (*y = ax + b)*. These lines minimize the sum of squared deviations between each point and the line.

Data were extracted from the database with *SAS Enterprise Guide 7.1* software*,* and analyses were performed with *SAS Enterprise Guide* and R version 3.6.3. We performed individual-based analyses and hospitalisation-based analyses.

#### Individual-based analyses

First, we graphically present (1) the annual number of people who are incarcerated hospitalised in psychiatric facilities (UHSAs, psychiatric hospitals, SMPRs, and maximum-security units) from 2009 to 2019; for each year, it was calculated as the number of incarcerated people present on January 1 of the year plus the number of incarcerated people entering a psychiatric facility during the year, (2) the annual number of nonincarcerated people hospitalised in psychiatric care per year in France from 2009 to 2019; for each year, it was calculated as the number of nonincarcerated people present on January 1 of the year plus the number of nonincarcerated people entering a psychiatric facility during the year, and (3) the number of incarcerated people on January 1 of each year, from 2009 to 2019. The annual percentage increase in the number of individuals compared to the number measured in 2009 was computed for the 3 populations.

Second, we present age, sex, and the principal ICD-10-coded diagnoses of incarcerated people hospitalised in psychiatric care. The principal ICD-10-coded diagnosis was collected for each stay; several primary diagnoses were thus possible for individuals with multiple hospitalisations. As a result, patients with multiple stays and diverging primary diagnoses were counted more than once for the analysis of diagnoses. We indicate the number of people with multiple diagnoses.

Third, we describe the number of people with 1, 2, 3, and 4 or more hospitalisations. We also present the distribution of the study population in the different psychiatric facilities (UHSAs, psychiatric hospitals, SMPRs, and maximum-security units) using a Venn diagram. The latter is based on all hospitalizations that occurred during the study period (2009–2019).

#### Hospitalisation-based analyses

First, we present the number of hospitalisations per year for people who are incarcerated according to the type of facility: UHSAs, psychiatric hospitals, SMPRs, and maximum-security units (UMDs) as well as the number of beds available in UHSAs. Importantly, when counting the number of stays per year, some overlapping stays were taken into account for consecutive years, resulting in a total per year higher than the number of stays for the study period (11 years). We also measured the following for the 2009–2019 period:

- annual rates of psychiatric hospitalisation among people who are incarcerated (i.e., the annual number of psychiatric hospitalisations divided by the number of people who are incarcerated on January 1);
- annual rates of psychiatric hospitalisation among people who are incarcerated for each facility (i.e., the annual number of psychiatric hospitalisations in each facility [UHSAs, psychiatric hospitals, SMPRs, maximum-security units] divided by the number of people who are incarcerated on January 1);
- annual rates of psychiatric hospitalisation among people who are incarcerated for daytime psychiatric hospitalisation (SMPRs) (i.e., the annual number of psychiatric daytime hospitalisations [SMPRs] divided by the number of people who are incarcerated on January 1);
- annual rates of psychiatric hospitalisation among people who are incarcerated for full-time psychiatric hospitalisation (i.e., the annual number of psychiatric full-time hospitalisations [UHSAs, psychiatric hospitals, maximum-security units] in each facility divided by the number of people who are incarcerated on January 1);

A Mann-Kendall Trend Test was performed to determine whether a trend existed in the four time series. The ratio of stays per patient was computed each year as the number of annual hospitalisations divided by the number of patients who had one or more stays during the year.

Second, we present the median (IQR) duration of hospitalisation in each facility. The length of stay for each hospitalisation was calculated as the date of admission to either the date of discharge or December 31, 2019, if the patient was still hospitalised. We also present the legal mode (voluntary, involuntary, or both) for hospitalisations in UHSAs. We describe the legal mode (voluntary versus involuntary) on admissions but also at discharge to identify hospitalisations that switched over in the middle.

Third, we focus on hospitalisations in UHSAs to determine the proportion of hospitalisations in these facilities directly preceded by a stay in another facility or directly followed by a stay in another facility.

### Role of the funding source

There was no funding source for this study. CCC had access to the PMSI database. All authors had access to aggregated data from this source as well as all other data used in the study. All authors decided to submit the manuscript for publication.

## Results

Between Jan 1, 2009, and Dec 31, 2019, 32,228 people who are incarcerated were hospitalised for psychiatric care (64,481 stays).

### Individual-based analyses

#### Longitudinal analysis of the number of incarcerated people hospitalised in psychiatric care

**Figure 1:**
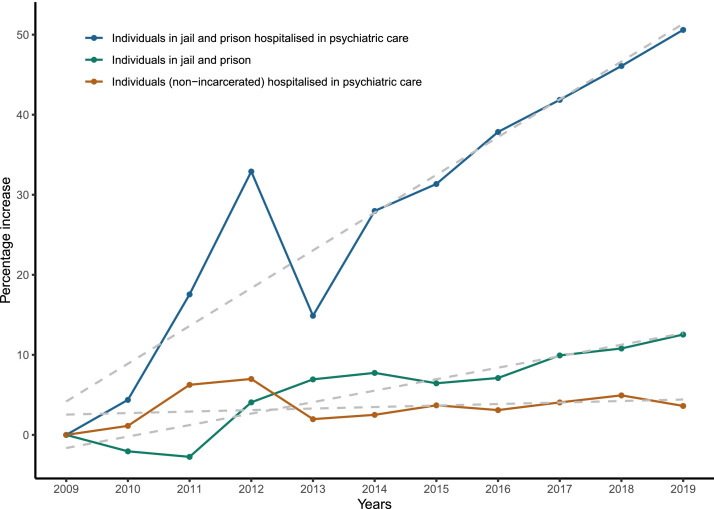
Annual percentage increase (compared to the number measured in 2009) in the number of (1) incarcerated people hospitalised in psychiatric facilities (in blue), (2) nonincarcerated people hospitalised in psychiatric care (in yellow), and (3) incarcerated people (in green) in France (2009–2019). *In all three plots, regression lines [E(percent increase in individuals) = beta_0 + beta_1(year)] are depicted as dashed lines.*

The number of incarcerated people hospitalised in psychiatric care per year in France ranged from 3263 to 4914 between 2009 and 2019 (see **Supplementary Fig. 1)**, i.e., an increase of 50.6% (see Figure 1). Over the same period, the number of prisoners and the number of nonincarcerated people hospitalised in psychiatric care per year increased from 62,252 to 70,059 (i.e., an increase of 12.5%) and from 405,117 to 419,794 (i.e., an increase of 3.6%), respectively (see Figure 1 and **Supplementary Fig. 1**).

#### Age, sex, and diagnoses

The age, sex, and diagnoses of the study population are presented in Table 2. among the 32,228 incarcerated people hospitalised in psychiatric care, 92.2% (*n* = 29,721) were men. The median age at the first hospitalisation was 32 years (IQR: 25–40), with 31 years (IQR: 25–40) and 34 years (IQR: 26–44) for men and women, respectively (*p* < 0.0001).

**Table 2:** Demographic characteristics and principal diagnoses of individuals in prison hospitalised in psychiatric care (2009–2019).

	Total *N* = 32,228	UHSA *n* = 10,619	PH *n* = 16,082	SMPR *n* = 12,785	UMD *n* = 826
Age (median, IQR)	32.0 [25.0–40.0]	32.0 [25.0;40.0]	31.0 [24.0;39.0]	31.0 [25.0;40.0]	31.0 [24.0;39.0]
Sex, Male (n,%)	29 721 (92.2%)	9 647 (90.8%)	15 022 (93.4%)	11,973 (93.6%)	808 (97.8%)
Sex, Female (n,%)	2 507 (7.8%)	972 (9.2%)	1 060 (6.6%)	812 (6.4%)	18 (2.2%)
*Principal diagnosis*					
**F00–99 Psychiatric disorder (n,%)**	2,6813 (83.2%)	9831 (92.6%)	14,164 (88.1%)	9889 (77.4%)	725 (87.8%)
F00–09 Organic mental disorders (n,%)	180 (0.6%)	88 (0.8%)	107 (0.7%)	51 (0.4%)	3 (0.4%)
F10–19 Mental and behavioural disorders due to psychoactive substance use (n,%)	3407 (10.6%)	859 (8.1%)	1478 (9.2%)	1991 (15.6%)	67 (8.1%)
F20–29 Schizophrenia, and delusional disorders (n,%)	8818 (27.4%)	4506 (42.4%)	5227 (32.5%)	2932 (22.9%)	463 (56.1%)
F30–39 Mood disorders (n,%)	4707 (14.6%)	2160 (20.3%)	2788 (17.3%)	1537 (12.0%)	89 (10.8%)
F40–48 Neurotic, stress-related and somatoform disorders (n,%)	6495 (20.2%)	2445 (23.0%)	3627 (22.6%)	2454 (19.2%)	75 (9.1%)
F60–69 Disorders of adult personality and behaviour (n,%)	7462 (23.2%)	3107 (29.3%)	4482 (27.9%)	2709 (21.2%)	263 (31.8%)
F70–79 Mental retardation (n,%)	331 (1.0%)	146 (1.4%)	204 (1.3%)	155 (1.2%)	13 (1.6%)
F90–98 Behavioural and emotional disorders with onset in childhood and adolescence (n,%)	389 (1.2%)	119 (1.1%)	238 (1.5%)	173 (1.4%)	15 (1.8%)
F99 Unspecified mental disorder (n,%)	519 (1.6%)	157 (1.5%)	355 (2.2%)	216 (1.7%)	27 (3.3%)

SMPR, services médico-psychologiques régionaux (daytime hospital psychiatric beds inside the prison); UHSA, unité hospitalière spécialement aménagée (full-time inpatient psychiatric ward exclusively for incarcerated people); UMD, unité pour malades difficiles (maximum-security psychiatric unit); PH, psychiatric hospital.

A psychiatric diagnosis was documented for 26,813 individuals (83.2%). There was no psychiatric diagnosis for 5415 individuals (16.8%). among them, the information was missing for 2063 individuals (6.4%), the codes Z00-Z99 (*factors influencing health status and contact with health services*) were used for 3112 individuals (9.7%), and other diagnoses were coded for 240 individuals (0.7%).

The most frequent principal psychiatric diagnoses were schizophrenia and delusional disorders (27.4%), disorders of adult personality and behaviour (23.2%), and neurotic, stress-related and somatoform disorders (20.2%). 18.0% of patients had multiple diagnoses.

#### Number and type of hospitalisations per patient

Most incarcerated people (61.4%, *n* = 19,799) had only one hospitalisation during the study period. The number (%) of people with 2, 3, and 4 or more hospitalisations was 6016 (18.7%), 2585 (8.0%) and 3828 (11.9%), respectively. The maximum number of hospitalisations for a single individual was 51. While most people stayed in only one type of facility, approximately one-fifth of patients were hospitalised in different (at least two) types of facilities (*n* = 6871, 21.4%). The exclusive distribution is described in **Supplementary Fig. 2**.

### Hospitalisation-based analyses

#### Longitudinal analysis of the number of hospitalisations according to the type of facility

Overall, 64,481 stays were analysed. Approximately one-third (28.2%) of the stays occurred in UHSAs (*n* = 18,187). Psychiatric hospitals, SMPRs, and maximum-security units (UMDs) accounted for 40.5% (*n* = 26,131), 31.1% (*n* = 20,066), and 1.6% (*n* = 1042) of the other stays, respectively.

**Supplementary Fig. 3** represents the number of stays per year according to each psychiatric facility (UHSAs, psychiatric hospitals, SMPRs, and maximum-security units). The number of stays in UHSAs increased from 300 (6.8% of the total number of hospitalisations) in 2010 to 3252 in 2019 (39.7% of the total). This increase corresponds to the progressive opening of these structures between 2009 and 2019 (the number of available beds in UHSAs is presented in Figure 3). In contrast, the number of stays in psychiatric hospitals and SMPRs remained constant (but the proportion of hospitalisations in these facilities decreased). The number of stays in maximum-security units (UMDs) remained constant.

**Figure 2:**
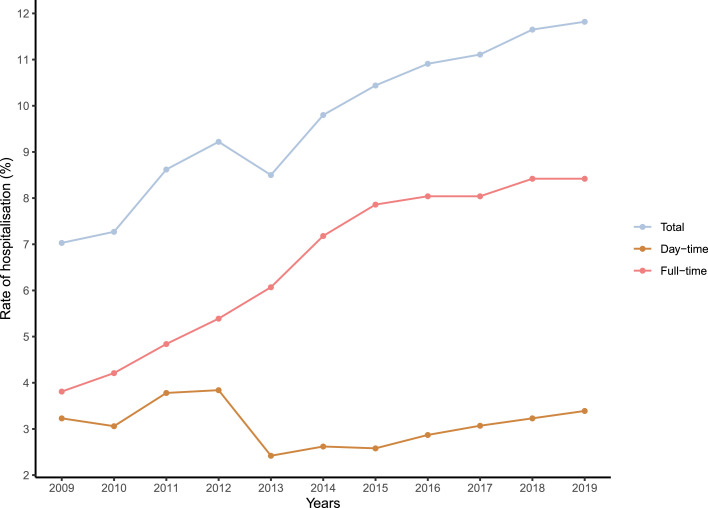
Annual rates of daytime (in yellow) and full-time (in pink) psychiatric hospitalisation (total, in blue) for people who are incarcerated between 2009 and 2019 in France.

**Figure 3:**
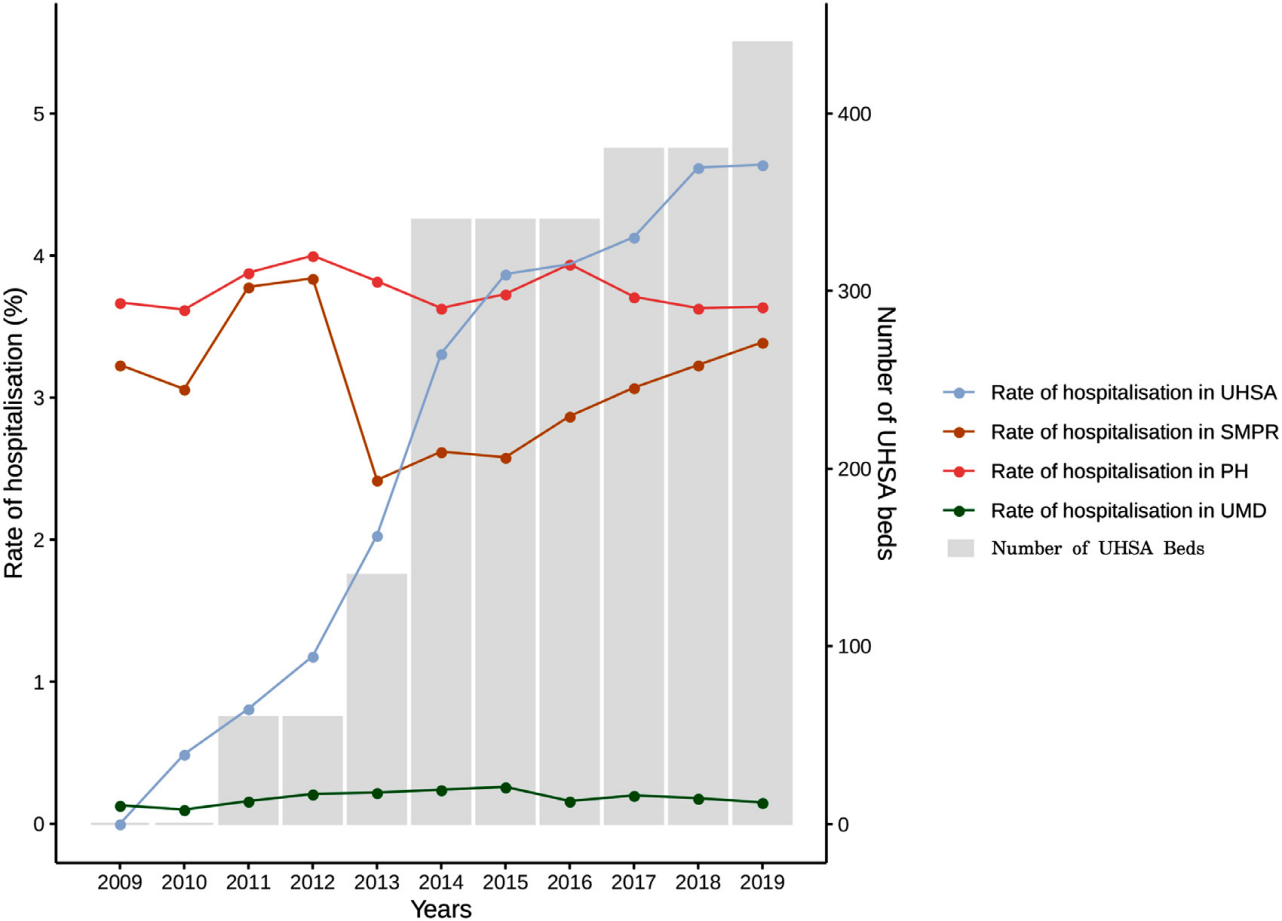
Annual rates of psychiatric hospitalisation for people who are incarcerated per type of facility (coloured curves) and the number of beds in UHSAs (grey bars) between 2009 and 2019 in France. *SMPR, services médico-psychologiques régionaux (daytime hospital psychiatric beds inside the prison); UHSA, unité hospitalière spécialement aménagée (full-time inpatient psychiatric ward exclusively for incarcerated people); UMD, unité pour malades difficiles (maximum-security psychiatric unit);* PH*, psychiatric hospital.*

Annual rates of hospitalisation (daytime hospitalisation, full-time hospitalisation, total) among people who are incarcerated are presented in Figure 2. Annual rates of hospitalisation for each psychiatric facility (UHSAs, psychiatric hospitals, SMPRs, and maximum-security units) are presented in Figure 3. While rates of hospitalisation in psychiatric hospitals, SMPR, and maximum-security units remained constant, rates of hospitalisation in USHAs continually increased. This was confirmed by a Mann-Kendall trend test, which reported a trend for UHSA (*p* < 0.001) and no trend for psychiatric hospitals (*p* = 0.061), SMPRs (*p* = 0.436), and maximum-security units (*p* = 0.310).

Between 2009 and 2019, the number of annual stays increased from 4358 to 8187, i.e., an increase of 87.86%, while the number of annual patients increased from 3263 to 4914, i.e., an increase of 50.60%. The number of stays increased faster than the number of patients, with the ratio of stay per patient increasing from 1.3 in 2009 to 1.7 in 2019.

#### Characteristics of the hospitalisations

The median duration of hospitalisation was 19 (IQR: 7–49) days. The median duration of full-time stays (UHSAs, psychiatric hospitals, and maximum-security units) and daytime hospital stays (SMPRs) were 14 (IQR: 6–34) and 37 (IQR: 14–104) days, respectively. The median duration of hospitalisation in UHSAs, psychiatric hospitals, and maximum-security units was 26 (IQR: 14–50) days, 8 (IQR: 4–20), and 54.5 (IQR: 8–233) days, respectively. Table 3 presents the characteristics of the hospitalisations included in the analysis.

**Table 3:** Characteristics of psychiatric hospitalisations for individuals in prison between 2009 and 2019 in France.

	Total	UHSA	PH	SMPR	UMD
Number of stays	64,481	18,187 (28.2%)	26,131 (40.5%)	20,066 (31.1%)	1042 (1.6%)
Median length of stay (median, IQR)	19 (7–49)	26 (14–50)	8 (4–20)	37 (14–104)	54.5 (8–233)

SMPR, services médico-psychologiques régionaux (daytime hospital psychiatric beds inside the prison); UHSA, unité hospitalière spécialement aménagée (full-time inpatient psychiatric ward exclusively for incarcerated people); UMD, unité pour malades difficiles (maximum-security psychiatric unit); PH, psychiatric hospital.

On admission to a UHSA, 64% of the stays (*n* = 11,638) were voluntary, and 36% (*n* = 6535) were involuntary. At discharge, 87.5% of stays (*n* = 15,917) were voluntary, and 12.5% (*n* = 2248) were involuntary.

#### Stays at UHSAs versus other facilities

Between 2009 and 2019, 3036 stays (16.7%) in UHSAs were directly preceded by a stay in another facility, with eight (0.3%), 2270 (77.8%), 700 (23.1%) and 58 (1.9%) from another UHSA, psychiatric hospitals, SMPRs and maximum-security units (UMDs), respectively. Of note, 1340 (7.3%) stays in the UHSA were directly followed by a stay in another facility, with eight (0.6%), 359 (26.8%), 908 (67.8%), and 65 (4.9%) in another UHSA, psychiatric hospitals, SMPRs and maximum-security units, respectively.

## Discussion

Developing systems for tracking the use of involuntary care and psychiatric hospitalisation in correctional contexts has recently been highlighted as a key factor for understanding the psychiatric needs of people who are incarcerated.^22^ In this study, we describe for the first time a longitudinal analysis of daytime and full-time psychiatric hospitalisations for individuals who are incarcerated in France between 2009 and 2019. Our results show that the rate of hospitalisation of people who are incarcerated substantially increased during this period. Importantly, the creation of new facilities exclusively dedicated to the full-time psychiatric hospitalisation of people who are incarcerated (UHSAs) in France did not reduce the hospitalisation of these people in psychiatric hospitals.

Linking the use of health care services with the need for care remains difficult. Nevertheless, the hypothesis of growth in the mental health needs of the prison population to explain the significant increase in the number of psychiatric hospitalisations for people who are incarcerated must be discussed. It is important to note that the overall prison population has also grown over this period, from 62,252 in 2009 to 70,059 in 2019. As the poor state of mental health in French prisons is well documented,^6^^,^^7^ this increase has probably been accompanied by an escalation in the need for psychiatric care in correctional facilities. However, while the prison population has “only” increased by 13%, the number of people in prison hospitalised in psychiatric care increased by 50% between 2009 and 2019. The increasing prison population cannot, therefore, be considered the sole explanation for the explosion in the number of psychiatric hospitalisations observed in our study. This period may have been marked by a deterioration in the mental health status of people in prison, although this has not been directly studied in our work. The increasing number of hospitalisations could also be due to an unmet need for psychiatric care, which has been filled by the increase in the number of beds. Particularly, the possibility for people who are incarcerated to be hospitalised voluntarily may have allowed access to psychiatric care for people who were not being treated before the beds were opened. The hypothesis of a demand induced by the development of UHSAs must also be considered. Based on the widely cited principle of Roemer's Law, which states that “*hospital beds that are built tend to be used*”,^23^ some studies have demonstrated a positive relationship between hospital bed availability and inpatient hospitalisation rates.^24^, ^25^, ^26^ However, some conflicting results have been shown regarding this principle.^27^^,^^28^ In addition, measuring the overall availability of hospital beds to a specific population is a complex task. Simply counting the number of beds is not sufficient, and it is essential to consider many factors, such as distance, demand, and access-related factors^26^; it was not possible to rigorously test these factors in our study.

Planning psychiatric hospitalisations of people in prison is challenging.^29^ On the one hand, the care provided to people who are incarcerated must be equivalent to that provided to the general population.^30^ On the other hand, legal and security-relevant aspects should be carefully considered.^31^ The development of UHSAs in France since 2010 aimed to meet these two objectives simultaneously. By allowing access to voluntary full-time psychiatric hospitalisation, which reached 64% of admissions in these facilities according to our study, the creation of UHSAs has contributed to optimizing the equivalence of care for people in prison. Importantly, these secure psychiatric hospitals were intended to accommodate people who are incarcerated suffering from psychiatric disorders under conditions that respected their rights.^32^ As mentioned above, the psychiatric hospitalisation of people who are incarcerated in community psychiatric hospitals is associated with several issues. In particular, the use of unsuitable premises with a high risk of escape sometimes leads to the use of isolation and mechanical restraint even when they are not clinically justified.^4^^,^^13^ Although the quality of care in each type of facility was not investigated in this study, the differences observed in lengths of stay may reflect the difficulty of providing optimal psychiatric care to people who are incarcerated in community psychiatric hospitals. Indeed, the median stay was 26 days in UHSAs compared with 8.8 days in psychiatric hospitals, whereas no major difference was shown in the diagnoses between the facilities, suggesting that the treatment of people who are incarcerated in psychiatric hospitals is limited to acute stabilisation. It could be hypothesized that inappropriate conditions for the full-time hospitalisation of people who are incarcerated in psychiatric hospitals lead psychiatrists to shorten the duration of stay in these facilities.^32^ Another important factor could be the pressure on the non-prison psychiatric system to discharge patients to free up beds. Importantly, our results show that despite the rapid increase in the number of UHSAs since their creation in 2010 (9 UHSAs, 440 beds), which has made it possible to accommodate more than 18,000 psychiatric stays, the number of stays in psychiatric hospitals and SMPRs remained constant during this period.

The persistence of a high number of people who are incarcerated admitted to psychiatric hospitals, despite the creation of UHSAs, is probably a consequence of several factors. First, it could be explained by an insufficient number of available beds (440) in these new structures. Indeed, if no bed is available in the neighboring UHSAs, the incarcerated patient is referred to the local psychiatric hospital, which could explain why the number of individuals who are incarcerated admitted to general psychiatric settings remained stable over the study period. This observation has led the French government to consider the construction of three new UHSAs (140 beds) in the coming years.^33^ Second, the referral of people in prison requiring inpatient psychiatric care to local psychiatric hospitals could also be related to the potential difficulties of access to UHSAs. Indeed, it has been recently shown that the UHSAs mainly accommodate patients from surrounding prisons^17^^,^^34^ and that the geographical distance makes it impossible to rapidly transfer individuals incarcerated in the most isolated prisons to UHSAs (in emergencies, people requiring full-time psychiatric hospitalisations are therefore referred to local psychiatric hospitals). Although this was not directly investigated in our study, it is important to note that we identified 3036 stays (16.7%) in UHSAs that were directly preceded by a stay in another facility. This type of transfer could reflect the difficulties of direct access to UHSAs. Third, the patients referred to UHSAs may have a clinical profile different than those referred to other facilities, but our results do not support this assertion, as the main diagnoses of patients in UHSAs do not differ significantly from those of patients admitted to other facilities.

Regarding daytime hospitals within prisons (SMPRs), their activity remained relatively stable between 2009 and 2019, apart from a slight transitory decrease at the time of the creation of the first UHSAs (2013). This probably reflects a reorientation of the type of psychiatric care within SMPRs with the opening of the UHSAs. Indeed, it can be hypothesized that the patients suffering from the most severe mental illnesses, frequently admitted to SMPRs before the creation of the UHSAs, were gradually redirected to these more appropriate care facilities. Another hypothesis is that this decrease, also observed for the number of people (not incarcerated) hospitalised in psychiatric care, could reflect a change in the PMSI data coding methods. Indeed, some psychiatric care activities were no longer considered hospitalisations after 2013. Unlike the UHSAs, the SMPRs are located within prisons and therefore allow the provision of psychiatric care without transferring the person who is incarcerated. However, no involuntary care is possible in these facilities because French law does not allow compulsory psychiatric treatment in prison.

Surprisingly, the number of incarcerated people hospitalised in maximum-security units (UMDs) remained low. Only 826 out of 32,228 patients were admitted to maximum-security units during the study period, from 84 stays in 2009 to 102 stays in 2019. Maximum-security units are facilities designed to care for the most difficult patients, especially those with a high risk of violence.^35^ The small number of people who are incarcerated could be explained by the difficulties in accessing these facilities. Indeed, the ten French maximum-security units can accommodate detained patients as well as patients transferred from psychiatric hospitals. Furthermore, as observed in our study, the median length of stay is higher in maximum-security units than in other facilities (although there are very few data available on this topic).

Several limitations of the present work should be acknowledged. First, we only had access to health data from the PMSI with limited sociodemographic data (only age and sex were available). No information about the severity of the index offence or the criminal history was available. Second, the use of administrative data does not allow for any confirmation of the diagnosis or the actual clinical status of the patient. However, our information is derived from diagnoses based on comprehensive clinical assessments by psychiatrists in hospital settings, and although diagnoses may vary among practitioners, high levels of agreement have been found for schizophrenia and bipolar disorders in France.^36^ Third, as our study was based on a single database, we cannot exclude variations in the quality of data coding over the years. Fourth, no outpatient mental health services data were available for the present study. Fifth, we only had access to French data. As a result, caution is needed before generalizing the results for other countries.

This work paves the way for many avenues of research to explain the trends observed in the present study. First, future studies should explore whether hospitalisations in USHAs are appropriate and investigate the quality of care in such facilities compared with psychiatric hospitals. Qualitative studies on care pathways would help to interpret the findings. Metrics such as suicide rates in prison could also be used to assess the impact of UHSAs on the mental health of people who are incarcerated. Second, we focused on hospitalisations for the present study, with little attention given to outpatient care, which is the mainstay of psychiatric treatment in both community and correctional settings. Access to outpatient care in French jails and prisons should be examined in future studies. Third, although it has not been rigorously tested, it seems that UHSAs accommodate more women proportionally (see Table 2). Future work should provide a better understanding of this finding. Finally, international comparisons regarding psychiatric hospitalisation among people who are incarcerated are urgently needed to help interpret the results presented in this study.

We conducted the first longitudinal study investigating the number of hospitalisations in psychiatry for people who are incarcerated in France. We showed that the number of people in prison hospitalised in psychiatric care increased drastically between 2009 and 2019 in France. Importantly, the creation of psychiatric hospitals specifically dedicated to this population (UHSAs) has not stopped the hospitalisation of people who are incarcerated in psychiatric hospitals. Further arrangements in the French justice system to optimize access to psychiatric care for people in prison will need to be developed and investigated in the future.

## Contributors

TF, CCC, MB and AL participated in the conception and design of the study; CCC participated in the acquisition of data; CCC, MB and AL performed the analyses and verified the underlying data; and TF wrote the first draft of the manuscript. All authors participated in the writing and revision of the successive drafts of the manuscript and approved the final version. All authors had full access to all the data in the study and accepted responsibility to submit for publication.

## Data sharing

This administrative data is only available through request from the French National System of Health Data (“Système National des Données de Santé”, SNDS), which manages this sensitive information (https://www.snds.gouv.fr/SNDS/Accueil), and cannot be shared.

## Funding

There was no funding source for this study.

## Declaration of interests

We declare no competing interests.

## Supplementary materials

Image, application 1
