# Supplementary material for: Psychiatric hospitalisations for people who are incarcerated, 2009–2019: An 11-year retrospective longitudinal study in France
Source: eClinicalMedicine. 2022 Apr 8;46:101374. doi: 10.1016/j.eclinm.2022.101374 (PMC9011007; doi:10.1016/j.eclinm.2022.101374)
Supplement: Supplementary file 1 [file mmc1.pdf]

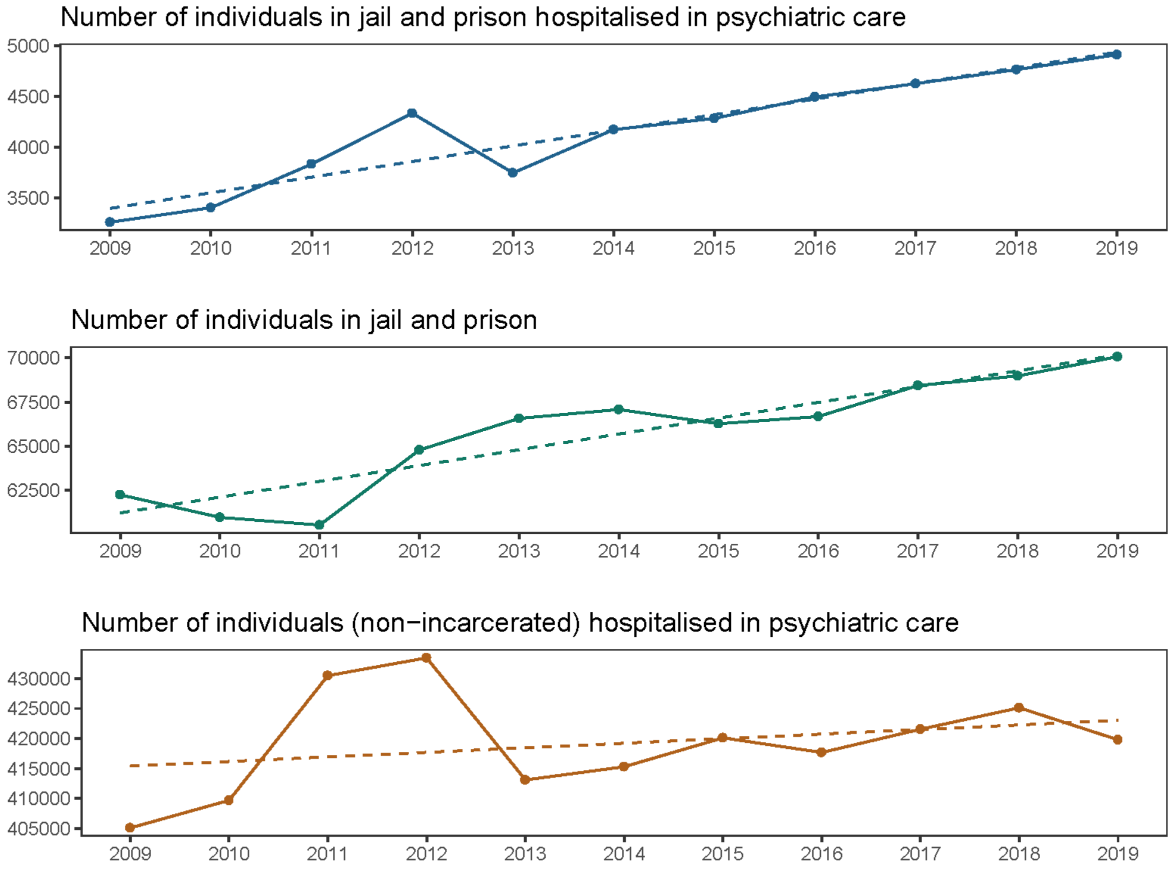

Supplementary Figure 1. Evolution of the number of incarcerated individuals hospitalised in psychiatric care, the number of individuals in jail and prison, and the number of individuals (nonincarcerated) hospitalised in psychiatric care per year in France (2009-2019)

In all three plots, regression lines are depicted in dashed lines.

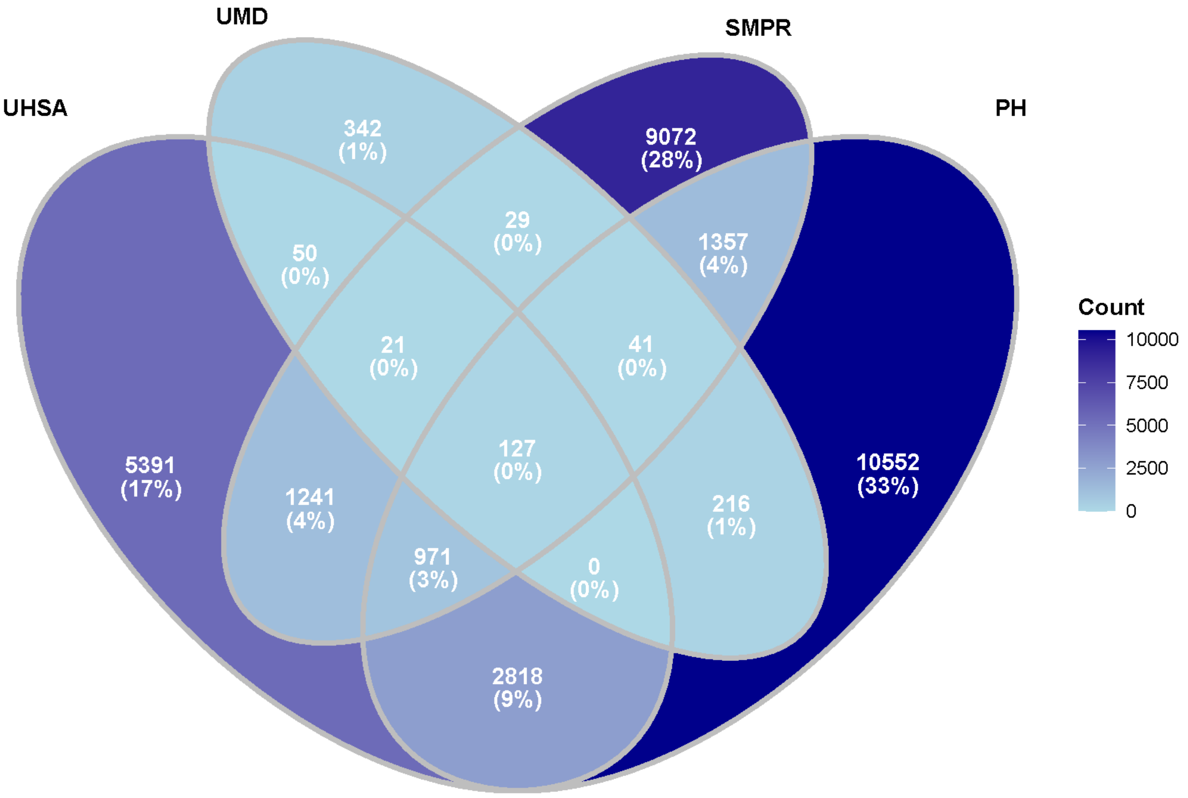

Supplementary Figure 2. Distribution of incarcerated people hospitalised for psychiatric care in France between 2009 and 2019 (N=32,228) in the different psychiatric facilities.

SMPR, services médico-psychologiques régionaux (regional medicalpsychological services); UHSA, unités hospitalières spécialement aménagées (specially equipped hospital units); UMD, unités pour malades difficiles (units for difficult patients); PH, psychiatric hospital.

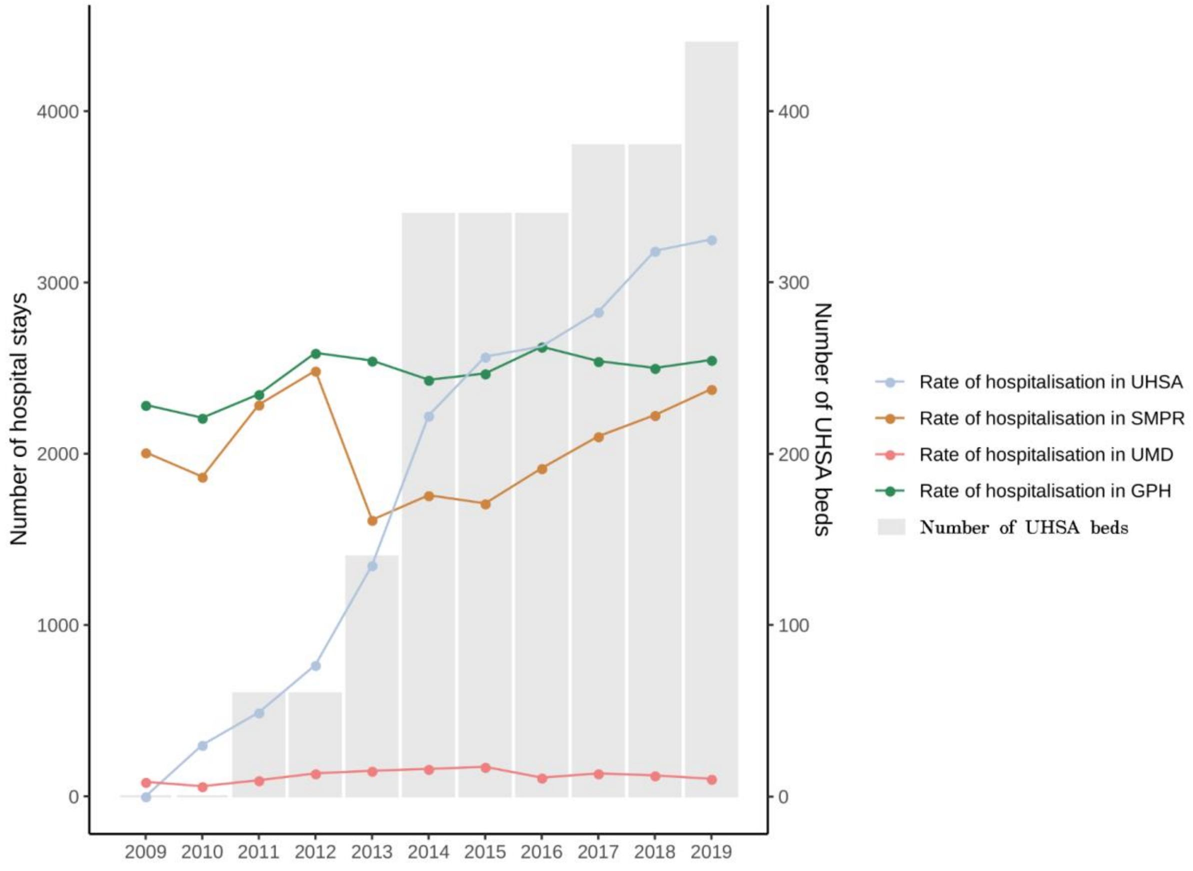

Supplementary Figure 3. Evolution of the number of psychiatric hospitalisations per type of facility (colored curves) and the number of beds in UHSAs (grey bars) between 2009 and 2019 in France.

SMPR, services médico-psychologiques régionaux (regional medicalpsychological services); UHSA, unités hospitalières spécialement aménagées (specially equipped hospital units); UMD, unités pour malades difficiles (units for difficult patients); PH, psychiatric hospital.
